# Stable-Isotope Dilution GC–MS Measurement of Metformin in Human Serum and Urine after Derivatization with Pentafluoropropionic Anhydride and Its Application in Becker Muscular Dystrophy Patients Administered with Metformin, l-Citrulline, or Their Combination

**DOI:** 10.3390/molecules27123850

**Published:** 2022-06-15

**Authors:** Svetlana Baskal, Alexander Bollenbach, Bettina Henzi, Patricia Hafner, Dirk Fischer, Dimitrios Tsikas

**Affiliations:** 1 Core Unit Proteomics, Institute of Toxicology, Hannover Medical School, 30623 Hannover, Germany; baskal.svetlana@mh-hannover.de (S.B.); bollenbach.alex@gmail.com (A.B.); 2 Division of Paediatric Neurology, University of Basel Children’s Hospital, 4056 Basel, Switzerland; bettina.henzi@ukbb.ch (B.H.); patricia.hafner@ukbb.ch (P.H.); dirk.fischer@ukbb.ch (D.F.)

**Keywords:** derivatization, metformin, GC–MS, pentafluoropropionic anhydride, serum, stability, urine

## Abstract

Metformin (*N*,*N*-dimethylguanylguanidine) is one of the most prescribed drugs with pleiotropic, exerted in part by not fully elucidated mechanisms of action. We developed and validated a gas chromatography–mass spectrometry (GC–MS) method for the quantitative analysis of metformin (metformin-d_0_) in 10-µL aliquots of human serum and urine using *N,N*-[*dimethylo*-^2^H_6_]guanylguanidine (metformin-d_6_) as the internal standard. The method involves evaporation of the samples to dryness, derivatization with pentafluoropropionic (PFP) anhydride in ethyl acetate (30 min, 65 °C), and extraction into toluene. The negative-ion chemical ionization GC–MS spectra of the PFP derivatives contain a single intense ion with mass-to-charge (*m/z*) ratios of *m/z* 383 for metformin-d_0_ and *m/z* 389 for metformin-d_6_. Our results suggest that all amine/imine groups of metformin-d_0_ and metformin-d_6_ are converted to their *N,N,N*-tripentafluoropropionyl derivatives, which cyclize to form a symmetric triazine derivative, of which the non-ring amine group is amidated. Quantification was performed by selected-ion monitoring (SIM) of *m/z* 383 and *m/z* 389. Upon validation, the method was applied to determine serum and urine metformin concentrations in 19 patients with Becker muscular dystrophy (BMD). Serum and urine samples were collected at baseline (Visit I), after six weeks of supplementation (Visit II) with metformin (3 × 500 mg/d; metformin group; *n* = 10) or l-citrulline (3 × 1500 mg/d; citrulline group; *n* = 9) followed by a six-week supplementation with 3 × 500 mg/d of metformin plus 3 × 1500 mg/d l-citrulline. At Visit I, the metformin concentration in the serum and urine was very low in both groups. The metformin concentrations in the serum and urine of the patients who first took metformin (MET group) were higher at Visit II and Visit III. The metformin concentration in the serum and urine samples of the patients who first took l-citrulline (CITR group) were higher at Visit III. The serum and urine concentrations of metformin were insignificantly lower in the CITR group at Visit III. The mean fractional excretion (FE) rate of metformin was 307% (Visit II) and 322% (Visit III) in the MET group, and 290% in the CITR group (Visit III). This observation suggests the accumulation of metformin in the kidney and its secretion in the urine. The GC–MS is suitable to measure reliably circulating and excretory metformin in clinical settings.

## 1. Introduction

**Scheme 1:**
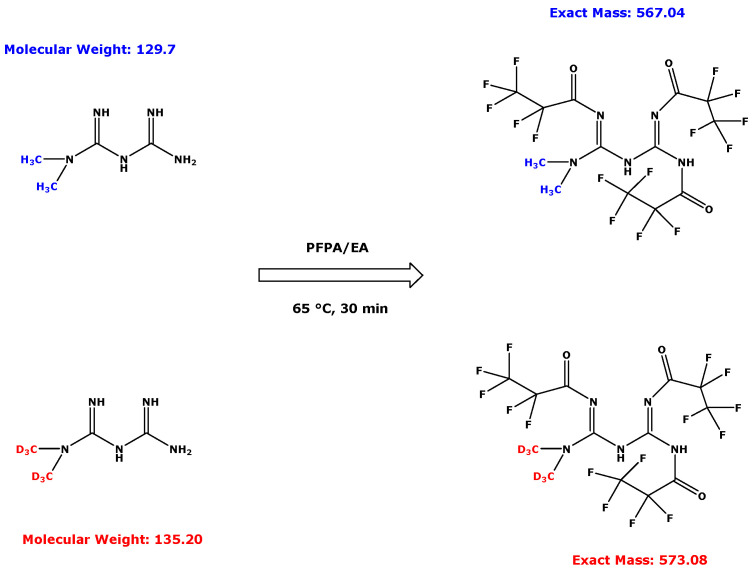
Chemical structures of unlabeled and deuterium-labeled metformin and their proposed derivatives upon derivatization with pentafluoropropionic anhydride (PFPA) in ethyl acetate (EA) (1:4, *v/v*).

The vast majority of analytes, including amino acids, drugs, and their metabolites, are not accessible to gas chromatography (GC)-based analyses, including gas chromatography–mass spectrometry (GC–MS), because they are not volatile and are thermally labile. This shortcoming can be solved by chemical reactions of functionalities, such as the carboxylic (COOH), amine (NH_2_ and NH), and hydroxyl (OH) groups with chemically reactive reagents to generate derivatives that are soluble in GC-compatible, water-immiscible organic solvents, electroneutral, volatile, and thermally stabile [1,2,3,4,5,6,7,8]. The anti-diabetic drug, metformin, i.e., *N*,*N*-dimethylguanylguanidine, is an asymmetrically dimethylated strong biguanide base (*p*K_a_, 12.4). Its amine and imine groups are accessible to derivatization (see Scheme 1). Heptafluorobutyric anhydride (HFBA) has been used for the derivatization and GC–MS analysis of metformin in biological samples after extraction [9,10]. The derivatization procedure, which was performed under reflux conditions for 1 h, was found to generate the metformin derivative 2-amino-4-dimethylamino-6-heptafluoro-*S*-triazine in the case of using HFBA [9,10]. This derivative still contains a non-derivatized amine group outside the aromatic ring. Amine and imine functionalities in methyl esters of amino acids can be amidated under milder derivatization conditions with pentafluoropropionic anhydride (PFPA) in ethyl acetate, for instance by heating at 65 °C for 30 min [8]. Under such conditions, it is expected that the three end-standing amine/imine groups of metformin are amidated with PFPA (Scheme 1). The aim of the present study was to test the utility of PFPA for the derivatization of metformin for its quantitative determination in human urine, serum, and plasma. It is noteworthy that metformin is not metabolized and is eliminated, unchanged by the kidney [11,12]. Indeed, we found that PFPA is suitable for the derivatization of metformin in biological samples. Here, we describe the development, validation, and application of a stable-isotope dilution GC–MS method for the measurement of metformin in the serum and urine samples obtained previously in a pilot study on Becker muscular dystrophy (BMD) patients who received metformin alone, l-citrulline alone, and their combination [13]. l-Arginine [14] and its precursor l-citrulline [15] may be beneficial to patients suffering from Becker muscular dystrophy (BMD) because they may increase endogenous nitric oxide (NO) production from l-arginine by means of neuronal NO synthase [13]. In the present study, we measured the concentration of metformin in urine and serum samples of the previous study [13].

## 2. Materials and Methods

### 2.1. Chemicals and Materials

Unlabeled metformin (metformin-d_0_), unlabeled creatinine (d_0_-creatinine), trideutero-creatinine, i.e., [*methylo*-^2^H_3_]creatinine (d_3_-creatinine; declared isotopic purity of >99 atom% ^2^H), and [*guanidino*-^2^H_6_]dimethylmetformin (declared isotopic purity of >99 atom% ^2^H) were purchased from Sigma-Aldrich (Steinheim, Germany). Pentafluoropropionic anhydride (PFPA) was from Thermo Scientific (Dreieich, Germany). Daily prepared solutions of PFPA in ethyl acetate (1:4, *v/v*) were used for the derivatization of metformin. Stock solutions were prepared in deionized water and stored in a refrigerator at 8 °C. Glassware for GC–MS (i.e., 1.5 mL autosampler glass vials and 0.2 mL microvials) and a fused-silica capillary column Optima 17 (15 m × 0.25 mm I.D., 0.25 µm film thickness) were purchased from Macherey-Nagel (Düren, Germany). 

### 2.2. Patients and Study Design

**Scheme 2:**
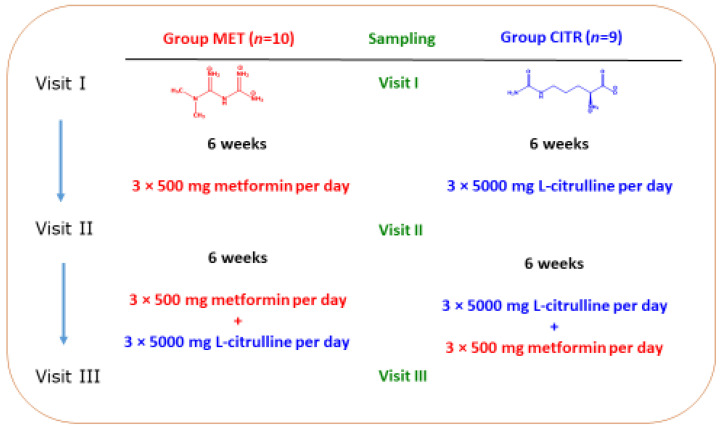
Study design on the effects of metformin and l-citrulline (for six weeks) and their combination (for six weeks) in patients with Becker muscular dystrophy (BMD). For more details, see the text.

The serum and urine samples analyzed in the present study originate as previously reported. Patients received metformin (MET group) or l-citrulline (CITR group) as the first treatments. Subsequently, both groups received a combination of the drugs. The study design is illustrated in Scheme 2. Blood and urine were collected immediately prior to the first treatment (Visit I, day 0). Patients were treated for six weeks either with 500 mg metformin (Sandoz Pharmaceuticals AG, Rotkreuz, Switzerland) thrice a day (MET Group) or with 5000 mg l-citrulline (l-citrulline drinking solution; Selectchemie Zuerich, Switzerland) thrice a day (CITR Group). At the end of this period, blood and urine samples were collected (Visit II, 6 weeks). Then, patients received both drugs at the same time, i.e., 3 × 500 mg metformin + 3 × 5 g l-citrulline for 6 weeks of treatment. Analogous, blood, and urine samples were collected immediately after the end of the combined medication (Visit III, week 12). The study was performed at the University of Basel Children’s Hospital (UKBB) as described previously in detail [13,14,15]. For the present study, serum and urine samples were available from 10 patients of the MET group and 9 patients from the CITR group for the measurement of metformin (Scheme 2).

### 2.3. Derivatization Procedure for Metformin in Human Serum and Urine Samples

For GC–MS analysis, metformin was derivatized with PFPA using a single derivatization step previously found useful for pentafluoropropionylation of amine and imine groups in methyl esters of natural amino acids, including various guanidine compounds and in polyamines [8,13].

For the generation of GC–MS spectra, aqueous solutions of synthetic metformin, i.e., metformin-d_0_ and metformin-d_6_, were placed in separate glass vials and the solvent was evaporated under a stream of nitrogen gas. The solid materials were then treated with 100-µL aliquots of a PFPA solution in ethyl acetate (1:4, *v/v*). The glass vials were tightly sealed, vortexed for 10 s, and the samples were heated for 30 min at 65 °C. After cooling to room temperature, the solvent and remaining PFPA were evaporated to dryness under a stream of nitrogen gas. The residues were treated first with 0.2 mL of toluene and then immediately thereafter with 200 µL of 0.4 M borate buffer, pH 8.5, to remove acidic compounds. The suspensions were mixed by vortexing for 2 min. Of the toluene phases, each 150-µL aliquot was decanted and transferred into an autosampler glass vial and capped tightly. Each 1-µL aliquot was drawn by the autosampler and injected splitless into the injector port. GC–MS spectra were generated by scanning the quadrupole in the mass-to-charge (*m/z*) range of 50–800 after negative-ion chemical ionization (NICI). 

### 2.4. Method Development and Validation for Serum and Urinary Metformin

The GC–MS method for metformin was validated in human urine and serum samples in relevant concentrations, i.e., as they were observed in chronic administration of metformin in anti-diabetic therapy [11,12]. Method validation included linearity, precision (expressed as relative standard deviation, RSD, %), and accuracy (expressed as recovery, %). Other aspects, such as metformin stability in urine and serum during storage and freeze/thaw cycles, were not considered because of the well-known chemical and metabolic stability of metformin in biological samples. The results of the validation experiments are reported in the Results section.

Urine and serum samples used in the method development and validation were obtained from BMD patients [13]. Prior to sample derivatization, urine and serum samples were thawed and centrifuged (5800× *g*, 5 min). Unspiked urine and serum samples (each 10-µL aliquots) and samples spiked with metformin-d_0_ and metformin-d_6_ were evaporated to complete dryness under a stream of nitrogen. Subsequently, derivatization was performed as described above (30 min, 65 °C). Organic solvents and excess PFPA were evaporated to dryness under a nitrogen stream. Then, the solid residues were reconstituted in borate buffer (200 µL) and followed by toluene (200 µL). The samples were then vortex-mixed for 60 s. Thereby, metformin derivatives were extracted into toluene, whereas the pentafluoropropionic acid formed from hydrolyzed and the reacted PFPA remained in the aqueous phase. After centrifugation (5 min, 4000× *g*), aliquots (150 µL) of the upper toluene phases were transferred into 1.5 mL autosampler glass vials equipped with 200 µL micro inserts. The samples were sealed tightly and subjected to GC–MS analysis.

### 2.5. Quantitative GC–MS Analyses of Metformin

A single-quadrupole mass spectrometer model ISQ directly interfaced with a Trace 1310 series gas chromatograph equipped with an autosampler AS 1310 from Thermo Fisher (Dreieich, Germany) was used in the GC–MS analyses. The interface, injector, and ion source were kept at 300 °C, 280 °C, and 250 °C, respectively. Helium was the carrier gas at a constant flow rate of 1 mL/min. Electron energy was set to 70 eV and the electron current to 50 µA. Methane (2.4 mL/min) was used as the reagent gas for negative-ion chemical ionization (NICI). The oven temperature was kept at 40 °C for 0.5 min, then increased to 210 °C at a rate of 15 °C/min and to 320 °C at a rate of 35 °C/min, respectively, and held at 320 °C for 1 min. Aliquots (1-µL) from toluene extracts were injected in the splitless mode. The autosampler was equipped with a 10-µL Hamilton needle, which was cleaned three times with toluene (5 µL) after each injection. 

Quantification was performed by select(ed)-ion monitoring (SIM) the ions with a mass-to-charge (*m/z*) of 383 for metformin-d_0_ and a *m/z* of 389 for metformin-d_6_. The dwell-time was 100 ms for each ion. The peak area (PA) values of metformin-d_0_ and metformin-d_6_ were calculated automatically by the GC–MS software (Xcalibur and Quan Browser). The concentration of metformin-d_0_ in the samples was calculated by multiplying the peak area ratio (PAR) of metformin-d_0_ to metformin-d_6_ with the known nominal concentration of the internal standard metformin-d_6_ added to the sample. Statistical analyses and graphs were performed and prepared by GraphPad Prism 7 (San Diego, CA, USA). Chemical structures were drawn by using ChemDrawProfessional 15.0 (Perkin Elmer, Waltham, MA, USA).

### 2.6. Calculation of the Fractional Excretion of Metformin

The fractional excretion rate of metformin ([FE_Metf_]) was calculated by using the Formula:

FE_Metf_ (%) = ([Metf]_U_ × [Creatinine]_S_)/([Metf]_S_ × [Creatinine]_U_) × 100

whereas [Metf]_U_ and [Metf]_S_ are the concentrations of metformin in the urine (U) and the serum (S) samples, respectively, and [Creatinine]_U_ and [Creatinine]_S_ are the concentrations of creatinine in the urine and the serum samples, respectively.

## 3. Results

### 3.1. GC–MS Characterization of Metformin-d_0_ and Metformin-d_6_ Derivatives 

GC–MS analyses of separate PFPA-derivatized metformin-d_0_ and metformin-d_6_ resulted in the formation (each) of a single intense GC–MS peak with a retention time of 3.67 min for the metformin-d_0_ derivative and 3.64 min for the metformin-d_6_ derivative. These retention times corresponded to an oven temperature of about 88 °C. We observed each of the three additional minor peaks (peak area, each less than 1% with respect to metformin at retention times of 5.25/5.20 min (peak I), 6.38/6.34 min (peak II), and 7.38/7.35 min (peak III)). The mass spectra of peak I contained the base peak, each at *m/z* 85. Low intensity paired ions were *m/z* 217/223, *m/z* 269/275, *m/z* 383/389 for peak I; *m/z* 245/251, *m/z* 259/265, *m/z* 383/389 for peak II, and *m/z* 365/371, *m/z* 383/389, *m/z* 413/419 for peak III. Peaks I, II, and III were not further considered. 

**Figure 1:**
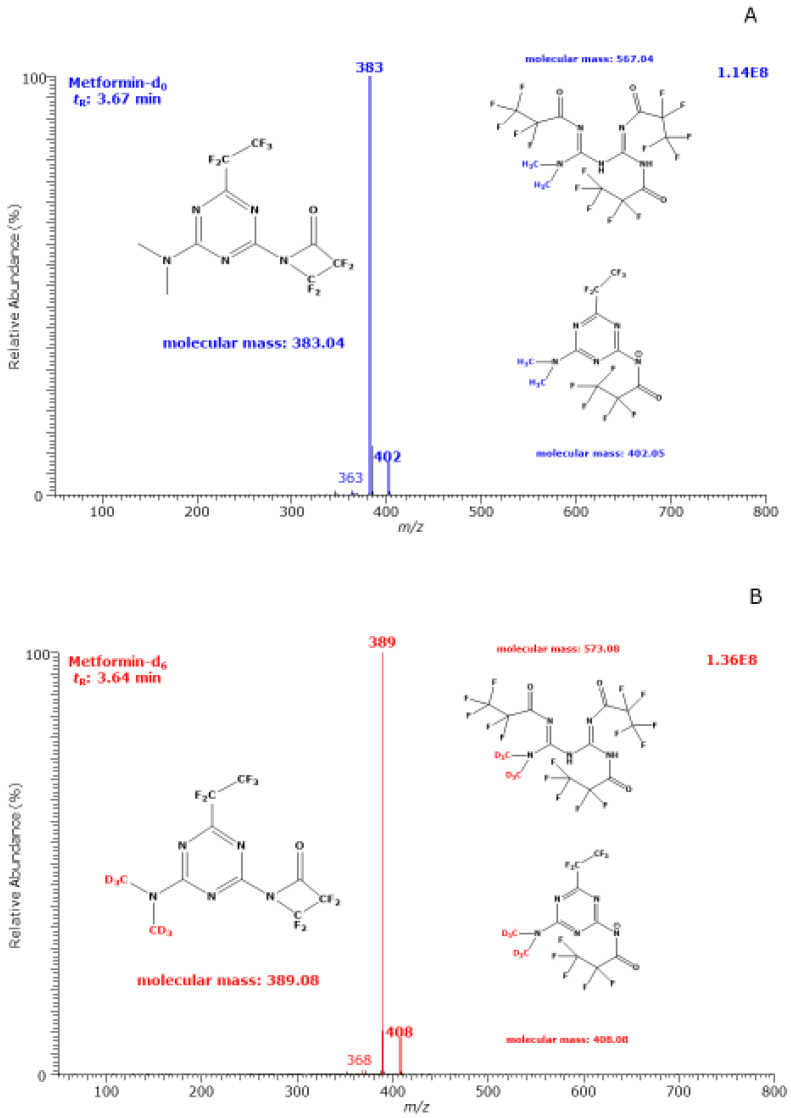
GC–MS spectra of (**A**) synthetic metformin-d_0_ (50 nmol) and (**B**) synthetic metformin-d_6_ (50 nmol) obtained by separate derivatization each of 100-µL aliquots of freshly prepared PFPA solution in ethyl acetate (1:4, *v/v*) and heating for 30 min at 65 °C. After extraction with toluene (1 mL), each 1-µL aliquot (corresponding to 50 pmol assuming a total yield of 100%) was injected and the negative-ion chemical ionization (NICI) mass spectra were acquired in the mass-to-charge (*m/z*) range 50–800 (1 scan/s). Inserts indicate the proposed structures of the intact derivatives and of the most intense ions *m/z* 383 and *m/z* 389.

We hypothesized that PFPA would react with the free imine/amine groups of metformin-d_0_ and metformin-d_6_ to form tri-PFP derivatives with molecular masses of 567 and 573, respectively (Figure 1). However, the largest ions in the GC–MS spectra of the peaks eluting at 3.67 and 3.64 min were *m/z* 383 and *m/z* 389. These ions could correspond to the triazine derivatives, of which, the non-ring amine group is also amidated with PFPA. The ions at *m/z* 383 and *m/z* 389 were likely to have been formed from the less intense ions at *m/z* 402 and *m/z* 408 by loss of F (19 Da) from the side chain of the triazine derivatives. Ions with lower *m/z* values were not observed, suggesting that *m/z* 383 and *m/z* 389 are very stable and do not further fragment. The difference of 6 Da strongly suggests that these ions bear the dimethylamine group. The GC–MS spectra suggest that metformin-d_0_ and metformin-d_6_ react with PFPA to form *N*-pentafluoropropionyl (PFP) derivatives with three PFP residues. 

**Scheme 3:**
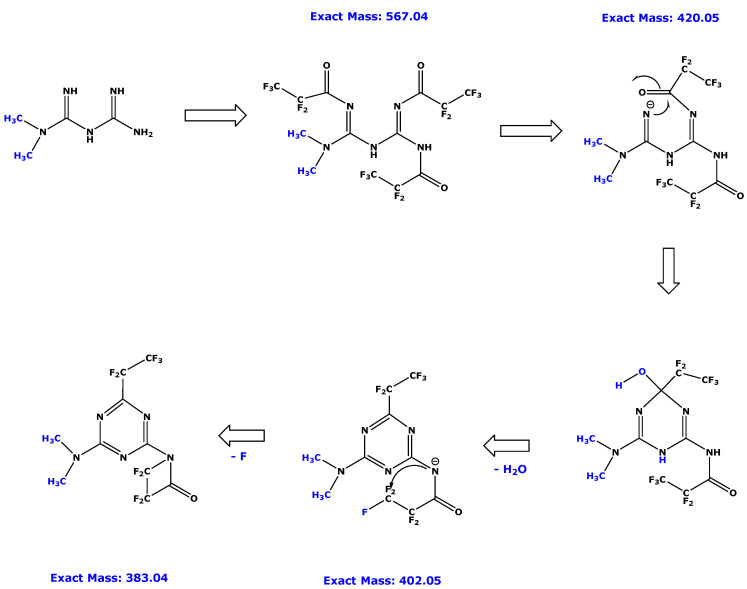
Proposed mechanism for the derivatization of unlabeled metformin with pentafluoropropionic anhydride (PFPA) in ethyl acetate (1:4, *v/v*) at 65 °C and 30 min and its ionization to form *m/z* 402 and *m/z* 383.

**Scheme 4:**
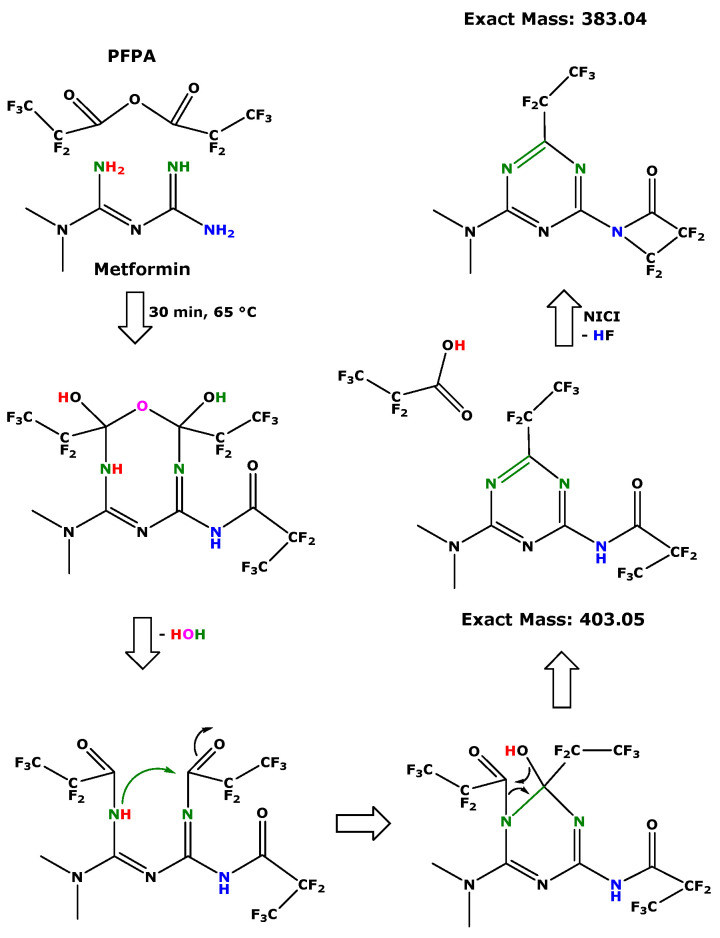
Proposal of an alternative mechanism for the derivatization of unlabeled metformin with pentafluoropropionic anhydride (PFPA) in ethyl acetate (1:4, *v/v*) at 65 °C and 30 min and its ionization to form *m/z* 402 and *m/z* 383. NICI, negative-ion chemical ionization.

Scheme 3 illustrates a possible mechanism for the formation of *m/z* 402 and *m/z* 383. The calculated molecular masses of 567 Da for metformin-d_0_-(PFP)_3_ and 573 Da for metformin-d_6_-(PFP)_3_ were not observed. This could be explained by further reaction during the derivatization process and/or due to fragmentation under the NICI conditions. The shorter retention time of metformin-d_6_-(PFP)_3_ compared to metformin-d_0_-(PFP)_3_ is due to the weaker interaction of deuterated compounds with stationary phases in chromatography. We did not observe any loss of deuterium atoms, suggesting that the *N*^G^-dimethyl group of metformin-d_6_ is stable during the derivatization process, its interaction with the stationary phase of the GC column, and the NICI of the derivative with methane as the reactant gas. Derivatization of metformin with HFBA and other anhydrides under refluxing conditions was reported to lead to triazine derivatives with a not-derivatized amine group [9,10]. An alternative mechanism that may lead to the same triazine derivative is the simultaneous reaction of a single PFPA molecule with the diamine groups of metformin at positions 1 and 5 (Scheme 4). The non-ring amine group is amidated by another PFPA molecule. Our present study suggests the formation of a triazine derivative of metformin with PFPA, of which the non-ring amine group is also amidated. In method validation, we performed quantitative analyses by SIM the ions with *m/z* 383 and *m/z* 402 for metformin-d_0_ and *m/z* 389 and *m/z* 408 for metformin-d_6_ for aqueous solutions of metformin-d_0_ (range, 0–200 µM) using a fixed concentration of 20 µM of metformin-d_6_. The retention times were 3.645 min (RSD, 0.14%) and 3.642 min (RSD, 0.19%) at *m/z* 383 and *m/z* 402, respectively. The retention times were 3.609 min (RSD, 0.08%) and 3.606 min (RSD, 0.14%) at *m/z* 383 and *m/z* 402, respectively. The peak area ratio (PAR) of *m/z* 389 to *m/z* 408 was 702:1 (RSD, 16%). The PAR of *m/z* 383 to *m/z* 402 was 670:1 (RSD, 12%). We observed linear relationships between the PAR of *m/z* 383 to *m/z* 389 against the concentration of metformin-d_0_ (*y* = 0.408 + 0.047 × *x*, *r*^2^ = 0.9886) and of the PAR of *m/z* 402 to *m/z* 408 against the concentration of metformin-d_0_ (*y* = 0.455 + 0.047 × *x*, *r*^2^ = 0.9739. The reciprocal value of the slope was 21.3 µM and close to nominal value of 20.0 µM metformin-d_6_. In quantitative GC–MS analyses in urine and serum samples, SIM of *m/z* 383 for metformin-d_0_ and *m/z* 389 for metformin-d_6_ was used for maximum specificity and sensitivity. 

### 3.2. Method Validation for the Quantitative GC–MS Measurement of Metformin in Human Urine and Serum Samples

Aliquots (10-µL) of aqueous solutions of metformin-d_0_ (11 concentrations in the range of 0–200 µM) and a fixed concentration of the internal standard metformin-d_6_ (20 µM) were subjected to derivatization with PFPA after evaporation to dryness as described above, and the toluene extracts were analyzed by GC–MS in the SIM mode. Plotting the peak area ratio (PAR) values of *m/z* 383 to *m/z* 389 (*y*) obtained in this validation experiment against the concentration of metformin-d_0_ (*x*) resulted in the regression equation *y* = 0.0496 × *x*, *r*^2^ = 0.9984. The reciprocal value of the slope is 20.2 µM metformin-d_6_, which is almost identical to the nominal metformin-d_6_ concentration of 20 µM in the samples. Plotting the metformin-d_0_ concentration calculated by multiplying the PAR measured by the concentration of 20 µM of the internal standard metformin-d_6_ resulted in the regression equation *y* = 0.9924 × *x* (*r*^2^ = 0.9984). The slope value of the equation indicates a mean recovery rate of 99.2% metformin.

A urine sample donated by a patient at Visit I was spiked with 1 mM metformin-d_6_ and varying concentrations of metformin-d_0_ (range, 0, 20, 40, 60, 80, 100 µM). Every two 10-µL aliquots of these samples were subjected to derivatization with PFPA as described above after evaporation to dryness. After derivatization and solvent evaporation, one series of the samples was extracted with toluene/borate buffer (experiment A) and the second series of samples was extracted only with toluene (experiment C). To 10-µL of aliquots of the above-mentioned spiked urine samples, each 90-µL aliquot of deionized water was added and 10-µL aliquots of the resulting dilutions were subjected to PFPA derivatization as described above, including complete solvent evaporation (experiment B). 

The retention times in these validation experiments (A, B, C) were determined to be 3.559 min (RSD, 0.13%) for the metformin-d_6_ derivative and 3.591 min (RSD, 0.06%) for the metformin-d_0_ derivative (*n* = 19). The peak area (in arbitrary units) of *m/z* 389 was 1.36 × 10^7^ (RSD, 3.1%) in experiment A, 1.56 × 10^6^ (RSD, 3.1%) in experiment B, and 1.01 × 10^7^ (RSD, 3.8%) in experiment C. These data indicate a robust GC–MS method for urinary metformin (see also below). The data of experiment (B) were used to estimate the limit of detection (LOD) of the method. The signal-to-noise (*S/N*) ratio of *m/z* 383 measured in the urine samples spiked with 2, 4, 6, 8, and 10 µM (6 ± 3.2 µM) was calculated by the software to be 5102 ± 1923. Considering that 10-µL urine samples were derivatized, the derivatives were extracted with 200 µL toluene, and 1-µL aliquots of the extracts were injected, it is calculated that the mean *S/N* value of 5102 corresponds to a mean metformin amount of 300 fmol. Extrapolation to an *S/N* value of 3:1 yields a mean LOD value of 176 amol metformin-d_0_.

**Figure 2:**
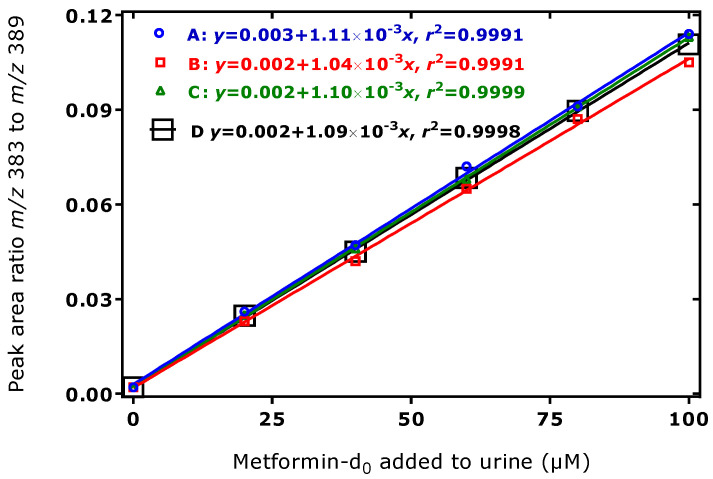
Relationships between the peak area ratio of *m/z* 383 to *m/z* 389 (*y*) and the metformin-d_0_ concentration added to a human urine sample. Three separate experiments were performed (A, B, C). In (D), the data of the individual experiments are combined. The nominal metformin-d_6_ concentration was 1000 µM. For more details, see the text.

The PAR values of *m/z* 383 to *m/z* 389 (*y*) obtained in this validation experiment were plotted against the concentration of metformin added to the urine sample (*x*). Linear relationships were obtained in the individual experiments A, B, and C (Figure 2). A linear relationship was also obtained when plotting the mean PAR against the added metformin-d_0_ concentration (data D in Figure 2). The precision (RSD) of the method when considering experiments A, B, and C ranged between 2.6% and 6.2%. The reciprocal values of the slope values of the regression equation were 897 µM in experiment A, 959 µM in experiment B, 903 µM in experiment C, and 919 µM in experiment D for metformin-d_6_; the calculated metformin-d_6_ concentration differs by 4–10% from the nominal metformin-d_6_ concentration of 1000 µM. The *y-axis* intercept of 0.002 indicates an apparent metformin-d_0_ concentration of 2 µM in the urine sample used in the validation experiments. These results are indicative of a precise and accurate method for the measurement of metformin in human urine in therapeutically relevant concentration ranges. 

**Figure 3:**
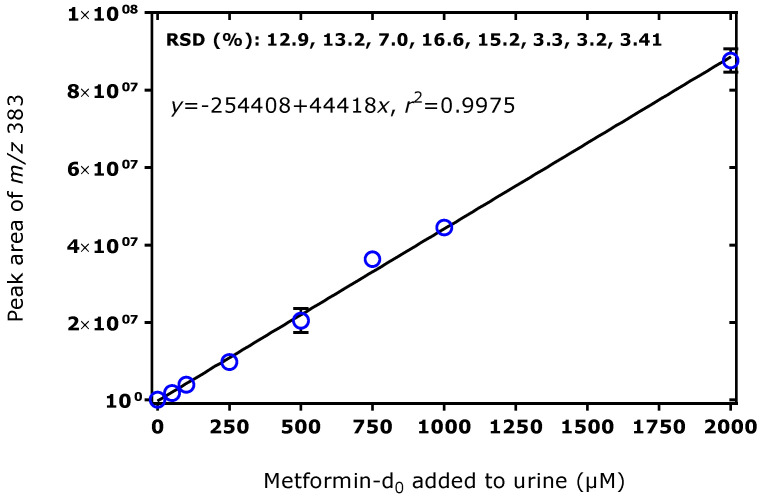
Relationship between the peak area of *m/z* 383 and the metformin-d_0_ concentration added to human urine. The internal standard metformin-d_6_ was not added to the urine samples. All three 10-µL aliquots of the human urine samples were spiked with metformin-d_0_ at the indicated concentrations and worked out as described in the text. Then, each 1-µL aliquot of toluene extract (200 µL) was injected and the mass fragment *m/z* 383 (see Figure 2 for A) for metformin-d_0_ was monitored. Data are shown as mean with the standard deviation. The inserted RSD values are listed consecutively in accordance with the added metformin-d_0_ concentrations.

We performed a metformin-d_0_ standard curve in a urine sample in the range of 0–2000 µM in triplicate, yet without the addition of the internal standard metformin-d_6_. Figure 3 indicates a linear relationship (*r*^2^ = 0.9975) between the peak area of *m/z* 383 and the concentration of metformin added to the urine. The peak area was measured with a precision (RSD) in the range of 3.2% to 16.6%. The peak area value obtained from the 2000 µM urine sample was 8.8 × 10^7^ a.u. These results suggest that the derivatization of urinary metformin with PFPA, the extraction, and the NICI of its derivative are quantitative, and the derivatives are stable. Thus, reliable quantitative determination of metformin in human urine could be possible without the use of the internal standard metformin-d_6_ or other external standards.

A serum sample donated by a patient at Visit I was spiked with 20 µM of metformin-d_6_ and varying concentrations of metformin-d_0_ (range, 0, 4, 8, 12, 16, 20 µM). Each 10-µL aliquot of these samples was subjected to derivatization with PFPA as described above after evaporation of the serum to dryness. After derivatization and solvent evaporation, the samples were extracted with toluene/borate buffer as described above for urine. A linear regression analysis between the metformin-d_0_ measured (*y*) and the metformin-d_0_ added (*x*) to the serum samples resulted in a straight line with the regression equation of *y* = −0.25 + 0.928 *x*, *r*^2^ = 0.9988, indicating a mean accuracy (recovery) of 92.8%. The serum sample spiked with metformin-d_6_ was only analyzed in triplicate with a precision (RSD) of 2.8%. These data indicate that metformin can be accurately and reproducibly measured in human serum samples at therapeutically relevant concentrations, such as in human urine samples.

**Figure 4:**
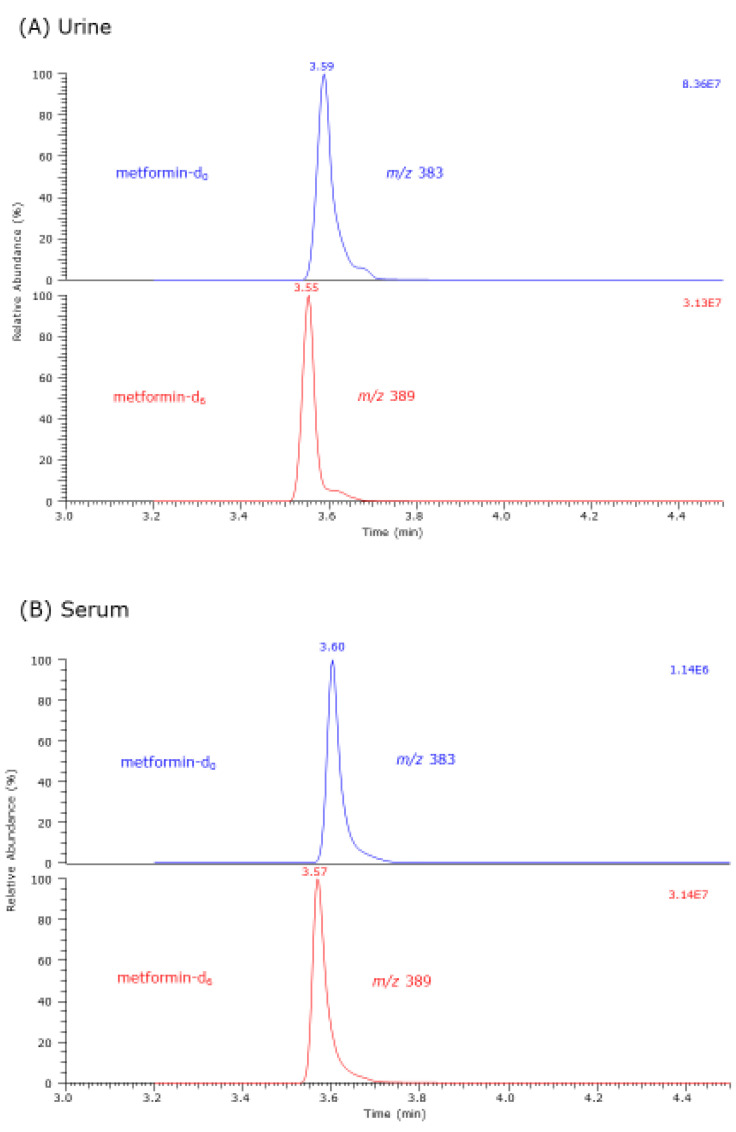
Partial GC–MS chromatograms from the quantitative analysis of metformin in (**A**) urine and (**B**) serum samples of Becker muscular dystrophy (BMD) patients. Selected ion monitoring of *m/z* 383 for metformin (metformin-d_0_) and *m/z* 389 for the internal standard (metformin-d_6_). The concentration of metformin-d_6_ was 100 µM in the urine and 20 µM in the serum sample.

Representative GC–MS chromatograms from quantitative analyses of metformin in human urine and serum samples using metformin-d_6_ as the internal standard are shown in Figure 4.

### 3.3. Serum and Urine Metformin Concentrations in the BMD Patients

**Figure 5:**
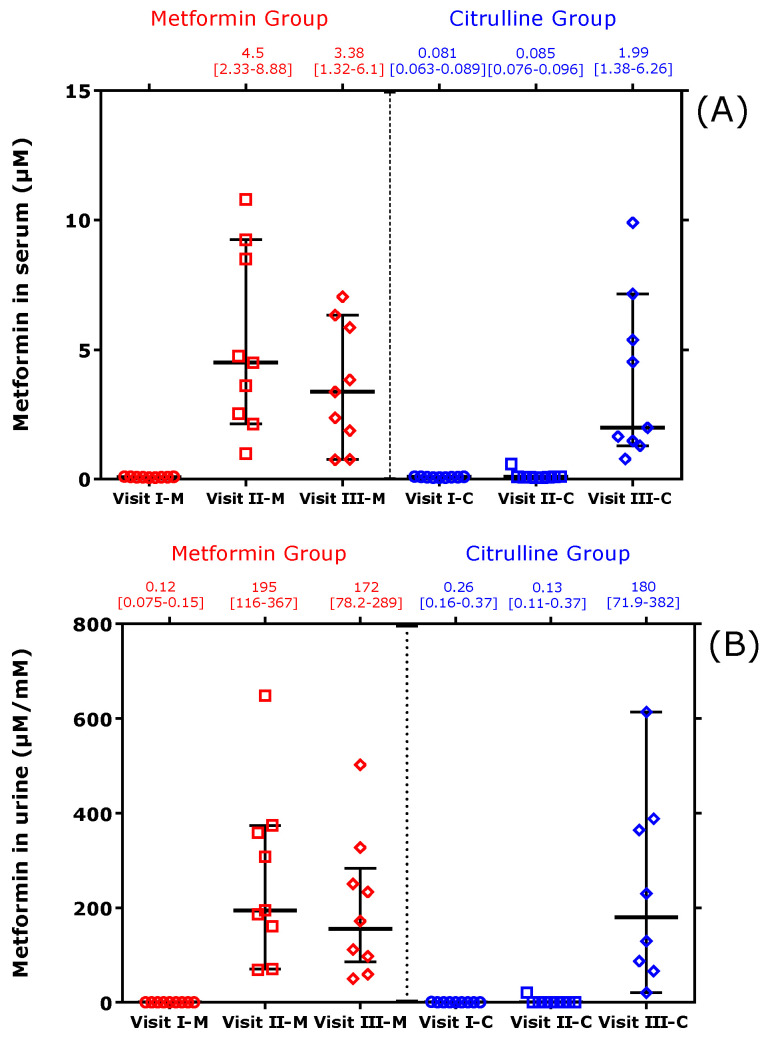
Concentrations of metformin in the serum (**A**) and of creatinine-corrected excretion rates in urine (**B**) of the BMD patients at baseline (Visit I) and after drug administration at Visit II and Visit III in the metformin (M) and citrulline (C) groups. Data are shown as median with 95% CI. Only statistical significances are shown (Mann–Whitney test). The concentrations of creatinine in the urine samples were measured by GC–MS and have been reported elsewhere [13].

Serum and urine samples were obtained and analyzed from 19 BMD patients—10 men from the MET group and 9 men from the CITR group—at three visits (Scheme 2). The metformin concentrations in the serum and urine samples of the patients of the MET and CITR groups measured with the present GC–MS method are shown in Figure 5. Correlations between serum and urine metformin concentrations were found at Visit III in the MET (*r* = 0.794, *p* = 0.009) and in the CITR (*r* = 0.905, *p* = 0.005) groups. Urinary metformin excretion and serum urea concentrations correlated only in the CITR group on Visit III (*r* = 0.762, *p* = 0.037). The metformin concentrations in the serum and urine differed in both groups when comparing Visit I with Visit III, as well as between the groups at Visits I, II, and III (Table 1).

These analyses were accompanied by quality control (QC) samples of pooled human plasma and urine samples or a healthy volunteer who did not ingest the drug metformin. No metformin was detected by the present GC–MS method in the QC plasma or urine. Metformin-d_0_ and metformin-d_6_ were each added to three 10-µL aliquots of plasma at concentrations of 0.5 mM (QC1), 1.0 mM (QC2), and 1.0 mM, respectively. In the QC1 and QC2 plasma samples, metformin-d_0_ was measured at 0.555 ± 0.010 mM (RSD, 1.27%) and 1.090 ± 0.000 mM (RSD, 0%), respectively. In the QC1 and QC2 urine samples, metformin-d_0_ was measured at 0.551 ± 0.004 mM (RSD, 0.64%) and 1.068 ± 0.004 mM (RSD, 0.33%), respectively. These data indicate that the metformin concentrations were measured in the serum and urine samples of the BMD with high accuracy and precision.

## 4. Discussion

Metformin (*N*,*N*-dimethylguanylguanidine) is a widely used anti-diabetic drug and possesses many pharmacological actions exerted in part by not fully elucidated mechanisms. Because of the great interest in metformin, several different analytical methods have been developed and used in clinical pharmacokinetic studies and clinical trials in the past decades [10,11,12,16,17,18,19,20,21,22,23]. GC-based methods have been among the first used for the analysis of metformin in biological fluids [16]. Recently, a GC–MS/MS method has been reported for the measurement of metformin in human hair [24]. The use of GC-based methods mostly requires (one or more) derivatization steps [1,2]. Monochlorodifluoroacetic anhydride (ClF_2_CH_2_-CO(O)-OC-CH_2_-F_2_Cl) has been used for the derivatization of metformin and its measurement in biological fluids by GC–MS and GC with electron-capture detection [9,16]. Metformin was found to form a 1,3,5-*S*-triazine derivative suggesting a biguanide cyclization [9]. Formation of an analogous triazine derivative was observed from metformin using HFBA (CF_3_CF_2_CF_2_-CO(O)-OC-CF_2_CF_2_CF_3_) [10]. Our study suggests that heating of metformin with PFPA at 65 °C for 30 min in ethyl acetate proceeds over the formation of a tri-PFP derivative as an intermediate, which cyclizes to form an 1,3,5-triazine analogous to monochlorodifluoroacetic anhydride [9,16] and heptafluorobutyric anhydride [10]. In contrast to previous methods, the derivatization of metformin with PFPA proceeds under less strong conditions and converts all amine/imine groups, including the non-ring amine group of the prospective triazine group into PFP amides. We suggest that the PFP groups at N1 and N3 undergo cyclization to form the symmetric triazine ring with the remaining non-ring moieties being CF_2_CF_3_ and COCF_2_CF_3_.

One-step derivatization of metformin with *N*-methyl-*bis*(trifluoroacetamide) (MBTFA) resulted in the formation of the molecular radical cation at *m/z* 303 for metformin-d_0_ and *m/z* 309 for metformin-d_6_ in the electron ionization (EI) mode [24]. The structure of this derivative was not elucidated in that study [24]. Previously, metformin was proposed by using GC–MS and EI to react with MBTFA to form a symmetric triazine derivative with CF_3_ and COCF_3_ as ring substitutes [25]. We did not directly compare MBTFA and PFPA in this study. Potential advantages/disadvantages of these derivatization reagents with respect to metformin measurement by GC–MS would be speculative. Whether PFPA allows for a more sensitive measurement in NICI because of its additional 4 F atoms compared to MBTFA remains to be demonstrated. 

The thermal stability of derivatives is of crucial importance in GC–MS and LC–MS analyses [26,27]. In previous investigations, we found that *N*-PFP derivatives of methyl esters of amino acids and their metabolites, including the guanidine compounds l-arginine, l-homoarginine, guanidinoacetate, and asymmetric dimethylarginine, form very thermally stable *N*-PFP derivatives in toluene [28]. In the present study, we did not explicitly investigate the thermal stability of the PFP derivative. Yet, we have no signs that the PFP derivative of metformin lacks the thermal stability that is required in GC–MS analyses. Our studies using PFPA show that *N*-PFP derivatives of amino acids are both thermally stable and remarkably stable against hydrolysis. 

The GC–MS peaks of the PFP derivatives of metformin-d_0_ and metformin-d_6_ show some tailing presumably due to the polarity of the stationary phase. However, this does not seem to affect the validity of the method. Moreover, this type of column allows reliable measurements of different kinds of derivatives of various classes of analytes, including amino acids, creatinine, nitrite, nitrate, and malondialdehyde [29]. Previously, we compared two GC columns of the same dimensions for the quantitative measurement of creatinine as the *N*-pentafluorobenzyl (PFB) derivative, i.e., Optima 17 and Optima δ6 [30]. According to the manufacturer (www.mnnet.com/tabid/5788/default.aspx), Optima δ6 possesses auto-selectivity. The PFB derivative of creatinine from human urine emerged as a symmetric peak from the Optima δ6 column and with a tailing from the Optima 17 column [30]. Yet, we found a very close correlation between the creatinine concentration measured in urine samples from 26 human subjects using these columns (*r* = 0.99974) and a close precision (0.92% and 1.12%, *p* = 0.25, respectively) [30]. It seems that the use of stable isotope-labeled analogs, such as metformin-d_6_, can fully compensate for potential problems arising from peak tailing.

Among the recently discovered actions of metformin is the enhancement of AMP-activated protein kinase activity in the skeletal muscles of subjects with type 2 diabetes [31]. The aim of the present study was to develop a GC–MS method for the determination of the concentration of metformin in urine and serum samples of the previous study [13]. Our results are in full accordance with the pharmacokinetics of chronically orally administered metformin to healthy and ill patients [10,11,12,16,17,18,19,20,21,22,23]. Much higher metformin concentrations were measured in the urine samples compared to the respective serum samples of patients who received metformin. Metformin is excreted unchanged in the urine. By using the respective serum and urine concentrations of metformin measured by the present GC–MS method and creatinine, we calculated the fractional excretion (FE) rate of metformin in the BMD patients. The mean FE rate of metformin was 307% (Visit II) and 322% (Visit III) in the MET group, and 290% in the CITR group (Visit III). This observation suggests that metformin accumulates in the kidney and is secreted in the urine [18], presumably via the organic cation transporter OCT2 in the kidney [32].

Our study indicates that GC–MS is suitable to measure reliably circulating and excretory metformin in clinical settings using a single derivatization step with PFPA and metformin-d_6_ as the internal standard. The method does not require the preceding extraction of metformin from urine and serum samples in contrast to other derivatization methods [9,10]. The extraction step with borate and toluene is useful for the elimination of organic acids, such as pentafluoropropionic acid, and PFP derivatives of polar substances, such as amino acids, which remain in the aqueous phase [8]. 

## 5. Conclusions

Pentafluoropropionic anhydride (PFPA) is a useful derivatization reagent for several classes of acidic, basic, and zwitterionic organic substances, including amino acids. The present study demonstrates that PFPA is also useful for the derivatization of metformin (*N*,*N*-dimethylguanylguanidine), a widely used anti-diabetic drug, in human urine and serum samples. PFPA reacts with all terminal amine/imine groups of metformin to form a symmetric triazine derivative, of which the non-ring amine group is also PFP amidated. The derivative is charge-free and readily soluble and stable in toluene. The GC–MS method is specific, accurate, precise, and sensitive. We measured the concentration of metformin in urine and serum samples of Becker muscular dystrophy patients administered with a therapeutic dose of metformin. The concentrations of metformin measured in the urine and serum samples are in accordance with the clinical pharmacokinetics of metformin. Our study suggests that metformin accumulates in the kidney and is secreted in the urine.

## Figures and Tables

**Table 1:** Summary of the results of statistical comparisons of metformin concentration in the serum and urine of the BMD patients of the metformin (M) and citrulline (C) groups at Visit I, Visit II, and Visit III.

Comparison	Serum	Urine
Visit I-M vs. Visit II-M	*p* = 0.0030 (paired *t* test)	*p* = 0.0020 (Wilcoxon test)
Visit I-M vs. Visit III-M	*p* = 0.0010 (paired *t* test)	*p* = 0.0020 (Wilcoxon test)
Visit II-M vs. Visit III-M	*p* = 0.3330 (paired *t* test)	*p* = 0.6031 (paired *t* test)
Visit I-C vs. Visit II-C	*p* = 0.2031 (Wilcoxon test)	*p* = 0.0273 (Wilcoxon test)
Visit I-C vs. Visit III-C	*p* = 0.0078 (paired *t* test)	*p* = 0.0078 (Wilcoxon test)
Visit II-C vs. Visit III-C	*p* = 0.0039 (Wilcoxon test	*p* = 0.0191 (paired *t* test)
Visit I-M vs. Visit I-C	*p* = 0.9597 (unpaired *t* test)	*p* = 0.0412 (Mann-Whitney test)
Visit II-M vs. Visit II-C	*p* = 0.0008 (Mann-Whitney test)	*p* = 0.0015 (unpaired *t* test)
Visit III-M vs. Visit III-C	*p* = 0.8026 (unpaired *t* test)	*p* = 0.8090 (unpaired *t* test)

## Data Availability

No data are available.

## Sample Availability

Not available.
